# Resolution of calcinosis using bisphosphonates in overlap syndrome – a case report

**DOI:** 10.1186/s41927-021-00176-5

**Published:** 2021-02-26

**Authors:** Mitchell Platter, Brian Pugmire, Reshma Patel

**Affiliations:** Department of Pediatric Rheumatology at Valley Children’s Hospital, 9300 Valley Children’s Place, Madera, CA 93720 USA

**Keywords:** Overlap syndrome, Juvenile dermatomyositis, Systemic lupus erythematosus, Calcinosis, Bisphosphonate, Alendronate, Case report

## Abstract

**Introduction:**

Calcinosis cutis is a common complication of pediatric rheumatologic diseases. However, there is currently no consensus on first-line treatment. Bisphosphonates have been described as a successful treatment in several case studies, but most of these cases are limited to patients with isolated juvenile dermatomyositis or systemic sclerosis. Specifically, there are limited reports of their usefulness in treating overlap syndromes and mixed connective tissue disorders.

**Case presentation:**

We describe the case of a 13 year-old girl with overlap syndrome with features of juvenile dermatomyositis and systemic lupus erythematosus. After 22 months of extensive immunosuppressive therapy, including monthly IVIG and Rituximab, she continued to have pain and weakness of the lower extremities. A CT scan was performed which showed significant multifocal soft tissue calcifications of the pelvis. She was started on treatment with oral alendronate with the goal of improving her calcinosis and improving her symptoms. After several months of therapy, our patient reported subjective improvement of her lower extremity pain and weakness, as well as complete resolution of abnormalities previously seen on physical examination. A repeat CT scan of the pelvis was performed after 11 months of therapy and demonstrated complete resolution of the previously seen calcinosis.

**Conclusions:**

We report the successful treatment of soft tissue calcinosis with oral bisphosphonates in a patient with juvenile dermatomyositis-systemic lupus erythematosus overlap syndrome. These results provide further evidence that bisphosphonates can be used successfully to treat calcinosis cutis in pediatric rheumatologic disorders. Additionally, the results provide new evidence that they can be used specifically in juvenile dermatomyositis-systemic lupus erythematosus overlap syndrome, which has not been previously reported.

## Background

Pediatric autoimmune connective tissue disorders (ACTD) encompass a wide range of diagnoses, each with their own unique symptoms and complications. One of the most common sources of morbidity amongst these diseases is subcutaneous calcinosis, which has been identified in 20–40% of cases of juvenile dermatomyositis (JDM) [1–3]. Calcinosis is most commonly identified in JDM and systemic sclerosis, but it can be seen in nearly all ACTDs [4]. The calcinosis in these disorders most commonly affects the knee joints, elbow joints, and hip processes [5]. Although it is a well-recognized source of morbidity in these disorders, there are currently no standardized recommendations for its treatment [3]. Of the ACTDs, treatment of calcinosis in JDM and systemic sclerosis has been most frequently reported, although there are currently no large scale, case-controlled studies. Rheumatologists often treat calcinosis with immunomodulating agents such as intravenous immunoglobulin (IVIG) and systemic glucocorticoids, or alternative agents such as bisphosphonates and calcium channel blockers [6]. Of these choices, bisphosphonates have been inconsistently associated with complete resolution of calcinosis in various case reports of patients with JDM [7, 8]. They are also noted to be relatively safe, with short-term side effects consisting of bone pain, hypocalcemia, and gastrointestinal symptoms such as diarrhea and abdominal pain. Longer-term use has been infrequently associated with impaired mineralization and nephrocalcinosis [9]. Although demonstrated to be effective and safe in treating calcinosis in JDM and systemic sclerosis, there is very limited data on the use of bisphosphonates in other rheumatologic disorders such as overlap syndromes and mixed connective tissue disorders (MCTD). In this review, we report the case of a 13 year-old girl with JDM-systemic lupus erythematosus (SLE) overlap syndrome who developed large, bulky calcinosis of the pelvis while on immunosuppressive therapy and demonstrated complete resolution after treatment with oral bisphosphonate therapy.

## Case presentation

We report the case of a 13 year-old, Hispanic girl who was diagnosed with JDM-SLE overlap syndrome at age 10. She initially presented to our hospital with complaints of symmetric upper and lower extremity muscle weakness and a photosensitive rash for 1 week prior to presentation. On physical examination, she had 3/5 strength in the proximal muscles of the bilateral upper and lower extremities, with an initial CMAS score of 6. Her cutaneous manifestations included a malar rash, heliotrope rash, and violaceous plaques on the dorsal aspect of the metacarpophalangeal joints consistent with Gottron papules. Her initial laboratory examination was significant for elevated muscle enzymes with an AST of 611, ALT of 392, alkaline phosphatase of 118, LDH of 1740, and creatine kinase of 16,012. She subsequently had a lower extremity MRI performed that showed diffuse, symmetric intramuscular T2 hyper intense signal within the gluteal adductor, extensor, and flexor musculature, consistent with diffuse proximal lower extremity myositis. On further laboratory examination, she was found to have a strongly positive ANA of 1:1280 with speckled pattern and a positive dsDNA by Crithidia of 19 (strongly positive > 9), with normal complements and negative Smith/RNP antibodies. With her clinical findings, laboratory evaluation, and radiologic findings she was diagnosed with a likely overlap syndrome with mostly features of JDM and SLE. As an inpatient, she was started on “protocol A” based on the “three consensus protocols for the initial treatment of moderate to severe juvenile dermatomyositis” [10]. She received IV methylprednisolone 30 mg/kg/day for 3 days and was started on subcutaneous methotrexate 15 mg/m^2 weekly. She was also started on oral hydroxychloroquine 200 mg/day, and after 3 days of methylprednisolone she was transitioned to oral prednisone 2 mg/kg/day divided BID. After the high dose steroids, her CK decreased to 5756 and her muscle strength improved as per PT evaluation, and she was discharged home 1 week after admission.

As an outpatient, she was scheduled to receive aggressive physical therapy. However, shortly after being discharged home she had two episodes of falling due to weakness and was readmitted 2 weeks after her initial presentation. She was found to have worsening proximal muscle strength at 2/5 in the lower extremities, a return of her photosensitive rash, and an increase in her CK to 8784. At this point she was switched to “Protocol B” of the above mentioned treatment recommendations and was given IVIG 2G/kg [10]. Given the severity of her weakness and her typical photosensitive rash, she was given another course of IV methylprednisolone 30 mg/kg/day for 3 days. She was then transferred to the inpatient rehabilitation unit where she completed 1 week of aggressive daily physical and occupational therapy. She was maintained on monthly IVIG and subcutaneous methotrexate along with a daily oral tapered prednisone regimen. She had another flare 11 months after her initial diagnosis which presented as a new inflammatory synovitis in the knee and wrist. She also had return of her malar rash, a worsening rash over her bilateral shoulders and chest (shawl sign), and elevation of her CK to 12,461, likely secondary to noncompliance with her outpatient IVIG infusions. She again received IV methylprednisolone 30 mg/kg/day for 3 days and IVIG 2G/kg. After discharge, despite better compliance with her monthly IVIG infusions, she continued to have episodes of weakness and elevated muscle enzymes with difficulty weaning off of prednisone. At 16 months after initial diagnosis, she was given two doses of Rituximab 750 mg/m^2 intravenous infusions for recalcitrant disease, and was maintained on IVIG 70 g every month. Two months after completion of her Rituximab course there was drastic improvement and eventually normalization of her serological muscle enzymes.

**Fig. 1:**
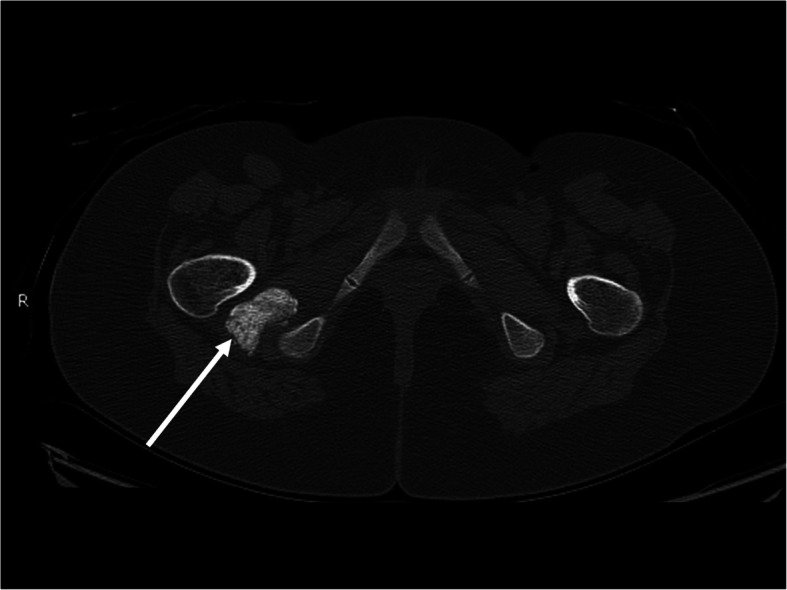
Axial CT image of the pelvis demonstrates a large area of bulky soft tissue calcification in the right ischiofemoral space (white arrow)

**Fig. 2:**
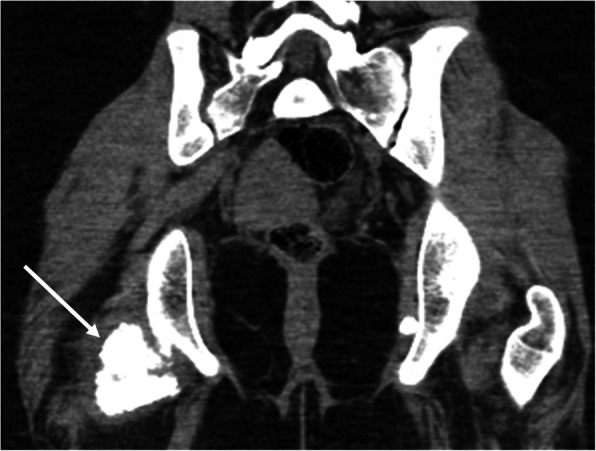
Coronal CT image of the pelvis from the same examination as Fig. 1 again shows the large area of soft tissue calcification in the right ischiofemoral space (white arrow)

**Fig. 3:**
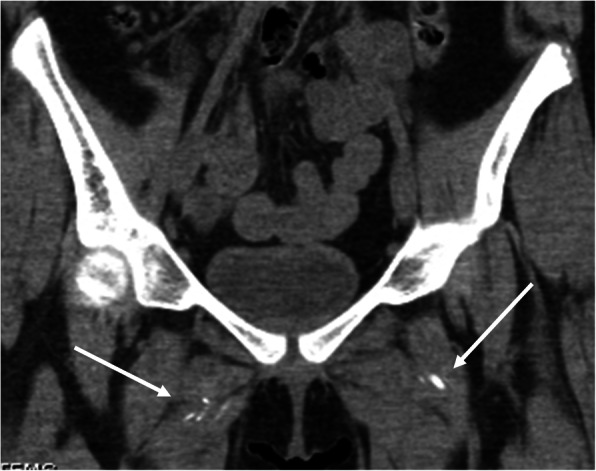
Coronal CT image from the same examination as Fig. 1 shows additional areas of soft tissue calcification in the right and left adductor musculature (white arrows)

At 22 months after diagnosis, she continued to complain of pain in her lower extremities despite receiving monthly IVIG and intense ongoing physical therapy. During an outpatient physical examination at this time, she was very hesitant to sit on the floor, and had to use Gower’s maneuver to stand from sitting. There was also new, decreased passive range of motion of the right hip when compared to the left. Estimated CMAS score at this time was improved to 47. Her muscle enzymes were also now persistently normal, so it was unclear if this was anatomical versus isolated muscle group weakness. Due to her continued symptoms and asymmetrical findings, an x-ray of the pelvis was ordered, which showed a rounded ossification near the right femoral neck, a smaller ossification near the left medial acetabulum, and multiple muscular calcifications in the adductor groups near the hip. A CT scan of the pelvis was ordered to further evaluate these findings, which showed a region of bulky, dense, soft tissue calcification measuring 3.8 × 2.3 cm within the ischiofemoral region on the right (Figs. 1 and 2), as well as scattered coarse linear calcifications within the abductor muscles bilaterally (Fig. 3). It also showed a 5.0 × 1.0 cm ovoid calcification in the left obturator internus muscle near the ischium. It was felt that these calcifications were contributing to her limitations in range of motion and subjective complaints, so additional therapy was pursued. She was started on the bisphosphonate alendronate orally at 10 mg/day in addition to her ongoing therapy with hydroxychloroquine, weekly methotrexate, and monthly IVIG.

**Fig. 4:**
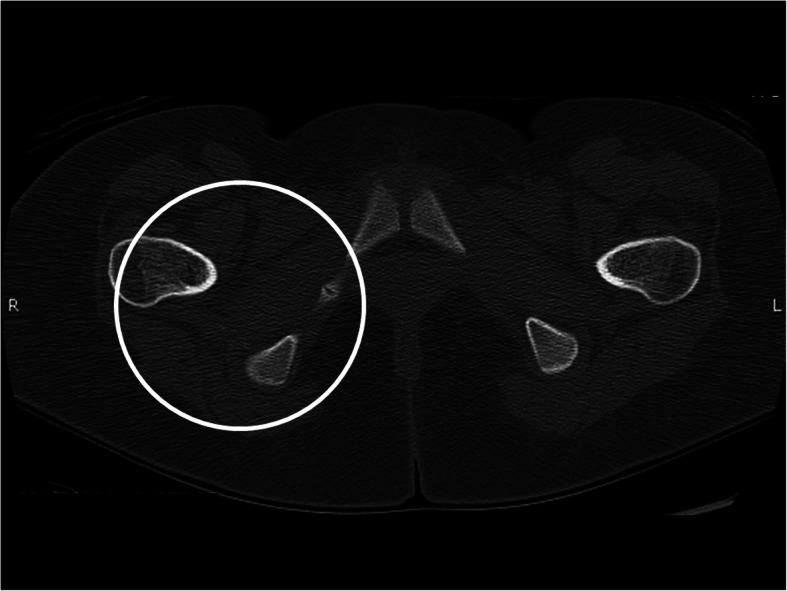
Axial CT image of the pelvis from a follow-up examination showed complete resolution of the area of bulky calcification (white circle)

**Fig. 5:**
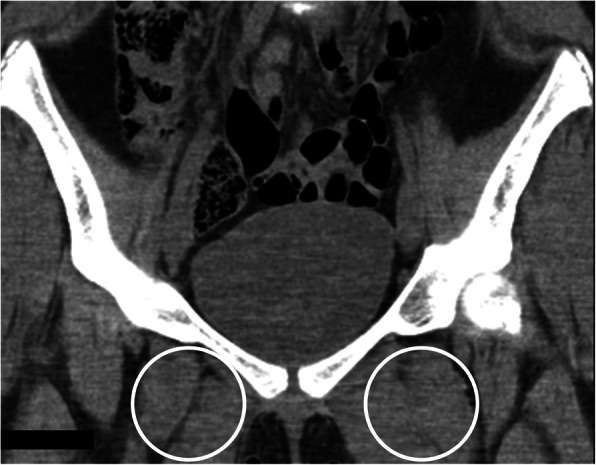
Coronal CT image from the same follow-up examination as Fig. 3 shows resolution of the areas of calcification in the adductor musculature (while circles

Our patient continued to follow up every 3 months in our clinic. Three months after starting alendronate she reported some improvement in her pain and strength, but on physical examination still had difficulty with squatting, and still used Gower’s maneuver when standing. A repeat x-ray was ordered at 7 months on bisphosphonate therapy, which showed complete resolution of previously seen calcified lesions. At her next follow up visit, after being on bisphosphonates for 10 months, she no longer had to use Gower’s maneuver with standing, and had equal range of motion in her bilateral lower extremities. A repeat CT scan was performed 11 months after therapy, which, similar to the x-ray, showed complete resolution of the previously seen soft tissue calcifications (Figs. 4 and 5). With complete resolution of the calcinosis, her alendronate was discontinued at her next follow-up visit. Throughout the duration of treatment, she did not report any of the short-term side effects such as GI irritation or new bone pain related to her bisphosphonate therapy.

## Discussion and conclusion

Our case demonstrates the successful use of oral bisphosphonates in resolving soft tissue calcinosis in a patient with JDM-SLE overlap syndrome. In the treatment of diseases causing excessive bone resorption such as osteoporosis, bisphosphonates work by inhibiting osteoclast function through various different mechanisms. Bisphosphonates have also been shown to inhibit the formation of calcium phosphate salts, thus making them a theoretical treatment option for disorders of excessive calcinosis such as JDM [11]. In previous case studies, it has been reported that bisphosphonates have been used successfully to treat calcinosis in patients with JDM [7, 8]. In one of the largest studies to date, six patients with JDM and calcinosis were all treated with bisphosphonates, and four of the six patients demonstrated complete resolution [7]. Additionally, in a large survey of pediatric rheumatologists, when asked about treating calcinosis in JDM, 73% said they would use bisphosphonates, and 60% ranked them as the most effective treatment [6]. Despite these studies, it is still unclear what the best option for treatment is, and there are currently no recommended guidelines for the treatment of calcinosis in JDM or other rheumatologic diseases. Our case provides further evidence that bisphosphonates can be an effective and safe medication used to treat calcinosis in rheumatologic diseases. Furthermore, while bisphosphonates have been reported as effective use in JDM, its use in other disorders is less widely reported. Our case demonstrates that bisphosphonates can be effective in rheumatologic diseases apart from JDM, in diseases such as overlap syndrome as seen in our patient. While our case report provides important new information, it is limited in the fact that it is a retrospective, observational report on a single patient. It would be beneficial to perform large, case-controlled studies in the future to further evaluate the overall effectiveness and safety of using bisphosphonates in the treatment of pediatric rheumatologic disorders.

## Data Availability

Data sharing is not applicable to this article as no datasets were generated or analyzed during the current study.

## Abbreviations

JDM: Juvenile dermatomyositis
SLE: Systemic lupus erythematosus
MCTD: Mixed connective tissue disorder
ACTD: Autoimmune connective tissue disorder
CMAS: Childhood myositis associated score
IVIG: Intravenous immunoglobulin
AST: Aspartate aminotransferase
ALT: Alanine aminotransferase
LDH: Lactate dehydrogenase
MRI: Magnetic resonance imaging
ANA: Antinuclear antibody
dsDNA: Double stranded DNA
RNP: Ribonucleoprotein
IV: Intravenous
CT: Computed tomography
